# Supplementary material for: Needle Tip Detection Using Ultrasound Probe for Vertical Punctures: A Simulation and Experimental Study
Source: Diagnostics (Basel). 2022 Feb 18;12(2):527. doi: 10.3390/diagnostics12020527 (PMC8871038; doi:10.3390/diagnostics12020527)
Supplement: Supplementary file 1 [file diagnostics-12-00527-s001.zip › diagnostics-1406553-supplementary/Manuscript-Supplementary/Supplementary.pdf]

diagnostics

Article

Needle Tip Detection Using Ultrasound Probe for Vertical Punctures: A Simulation and Experimental Study

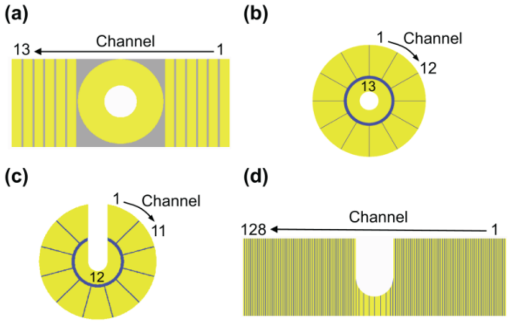

Figure S1. Four types of ultrasound array probes for vertical puncture: (a) 13-element linear array ultrasound probe with through-hole in the center (type A) [21]; (b) 13-element ring-type array ultrasound probe (type B) [20]; (c) U-type slit ring-type array ultrasound probe (type C); and (d) 128-element linear array probe (type D) [21]. (a) and (d) are Copyright (2014) The Japan Society of Applied Physics.

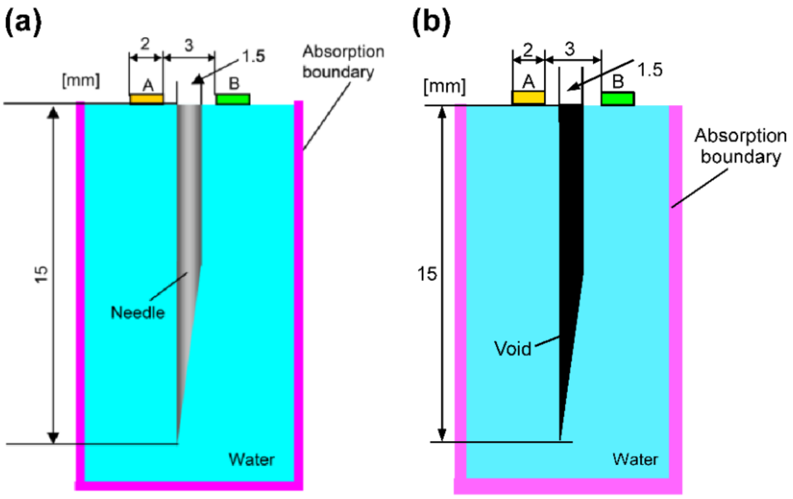

Figure S2. Needle simulation model [21]: (a) stainless steel needle and (b) void needle. (a) is Copyright (2014) The Japan Society of Applied Physics.

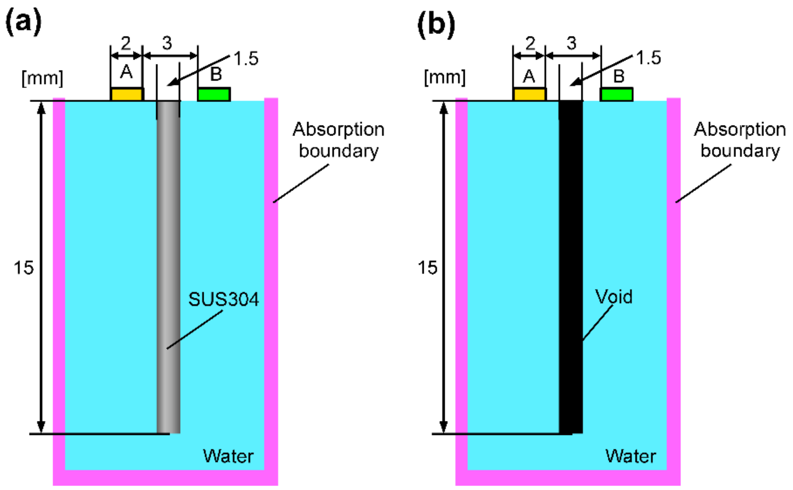

Figure S3. Cylinder simulation model: (a) stainless steel cylinder and (b) void cylinder.

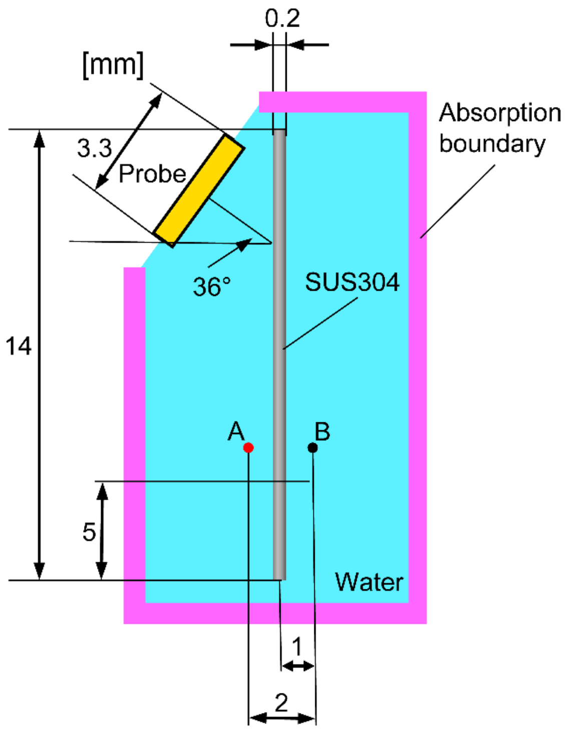

Figure S4. Ultrasound-guided wave simulation model.

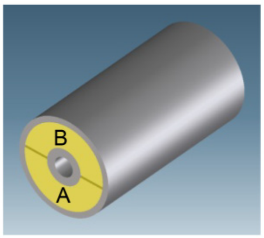

Figure S5. Ultrasound probe composed of separated transducers [20].

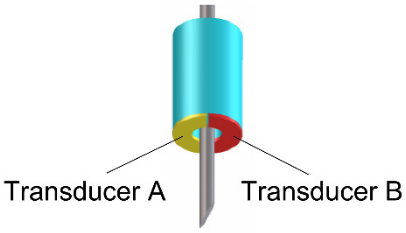

Figure S6. Configuration of the needle slope.

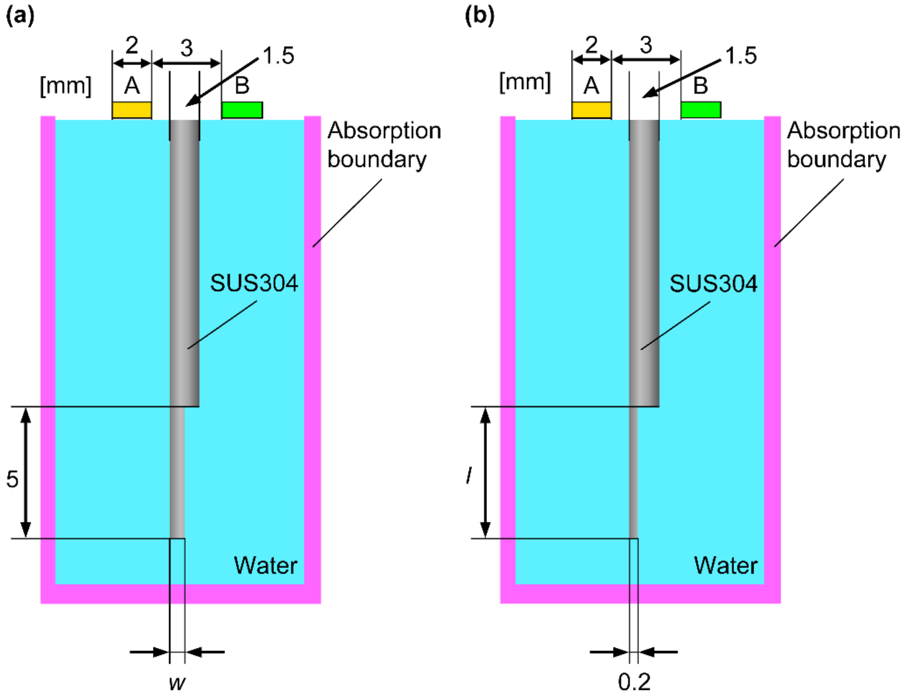

Figure S7. Simulation models for needle tip detection: (a) cylinder tip width w is 0.1–1.0 mm and (b) cylinder tip length l is 1–10 mm.

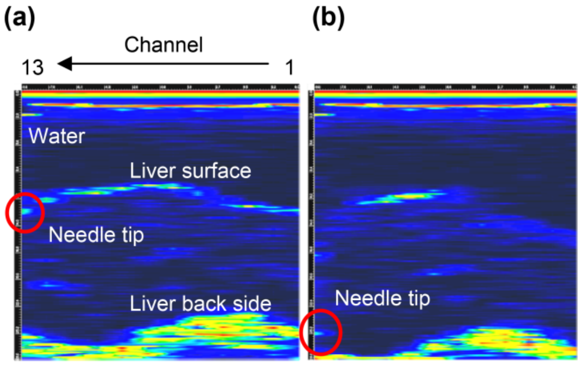

Figure S8. B-mode images of the needle tip and porcine liver using a 13-element ring-type array probe (Type B): (a) needle tip near the surface of the liver and (b) needle tip near the back of the liver [20].

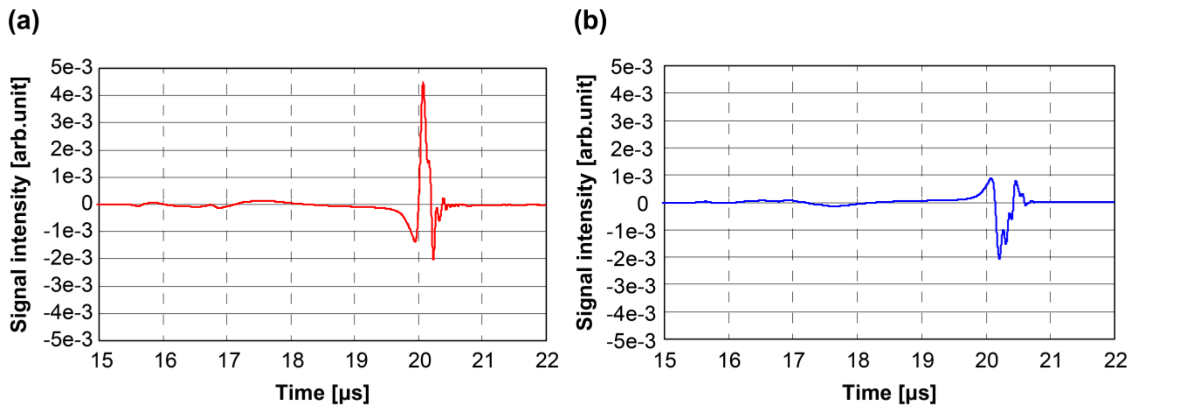

Figure S9. Waveforms from the needle tip in the simulation: (a) at A-side receiver and (b) at B-side receiver.

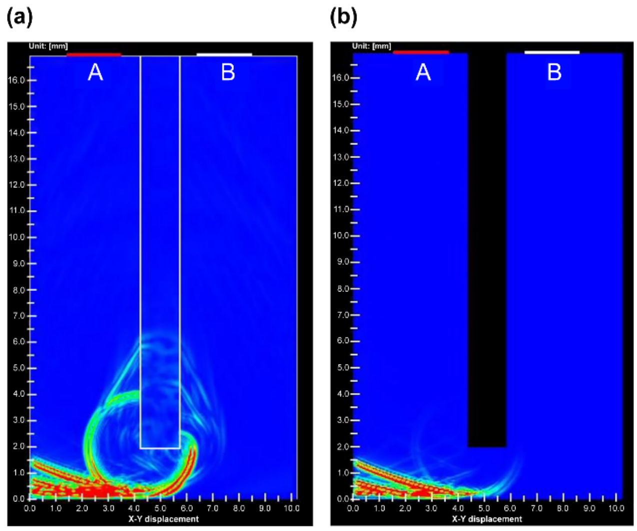

Figure S10. Simulation image of ultrasound propagation at the tip of the cylinders: (a) stainless steel cylinder and (b) void cylinder.

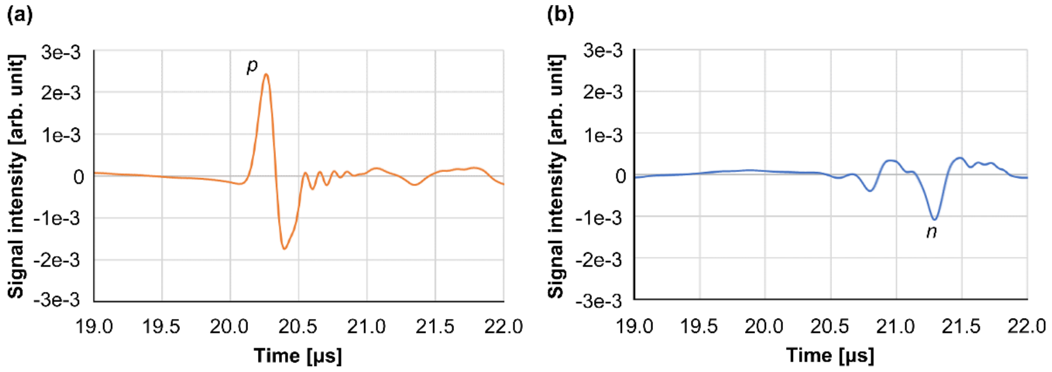

Figure S11. Waveforms from the stainless cylinder in the simulation: (a) A-side receiver and (b) B-side receiver.

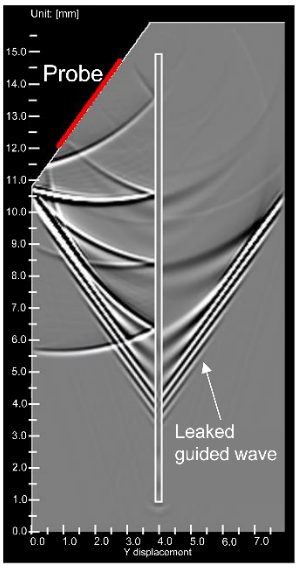

Figure S12. Ultrasound propagation of guided wave: frequency is 7 MHz, and white indicated positive amplitude, whereas black indicated negative amplitude.

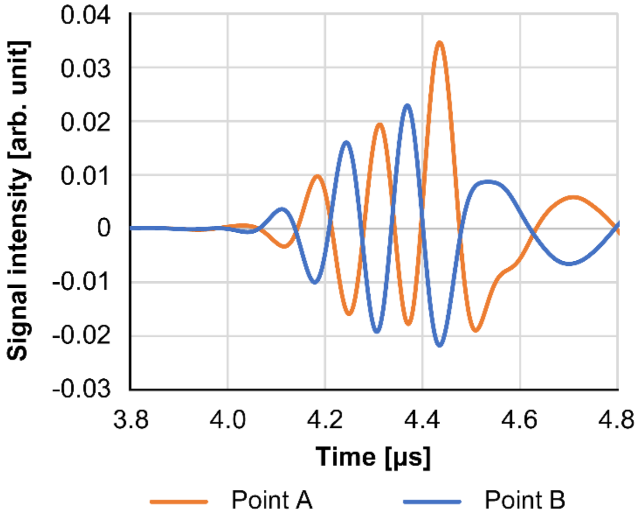

Figure S13. Waveform of leaked ultrasound-guided wave at points A and B in Figure S4.

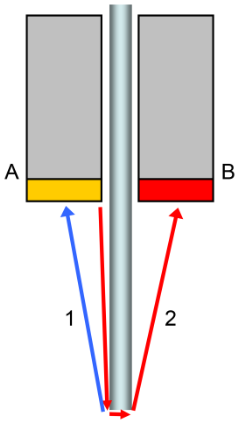

Figure S14. Propagation paths for the cylinder [26]. Red and blue arrows indicate inverse phases.

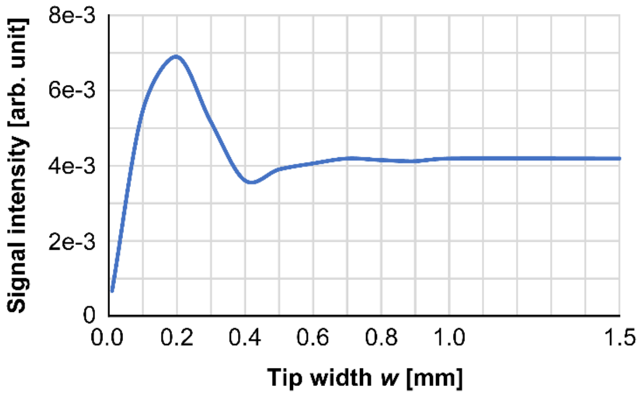

Figure S15. Relationship between tip width and signal intensity.

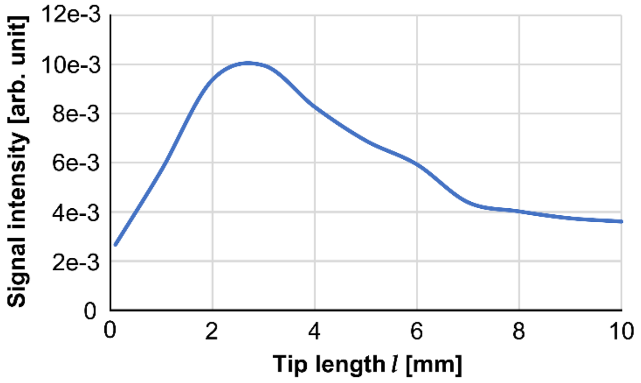

Figure S16. Relationship between tip length and signal intensity.

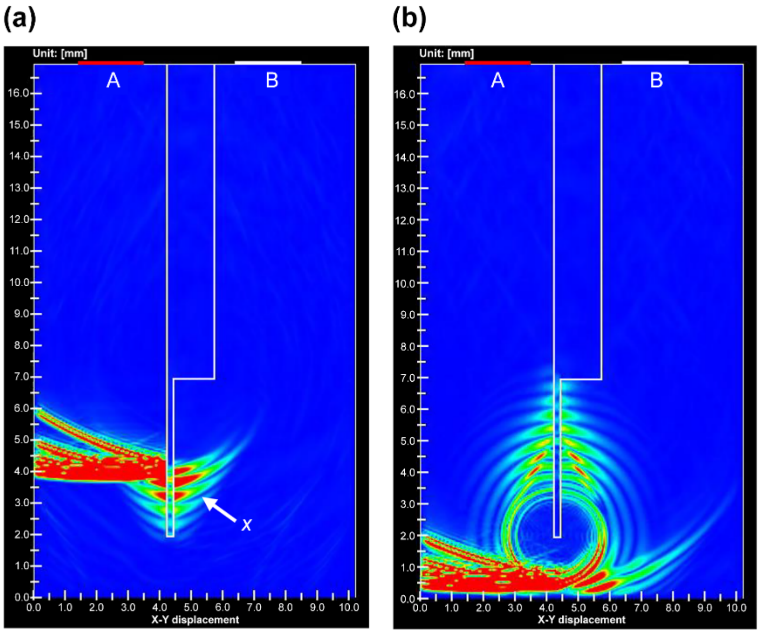

Figure S17. Ultrasound propagation at a 0.2-mm tip width: (a) before reflecting at the tip and (b) after reflecting at the tip.

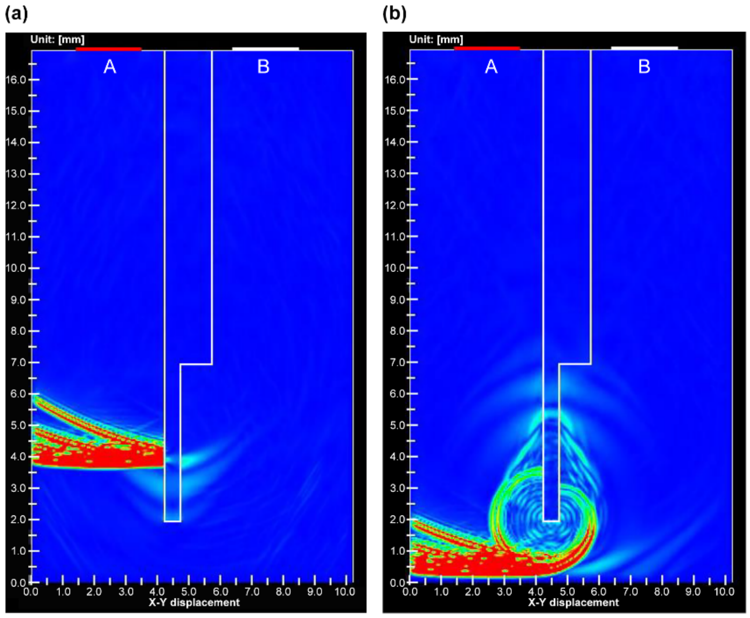

Figure S18. Ultrasound propagation at a 0.5-mm tip width: (a) before reflecting at the tip and (b) after reflecting at the tip.
