# Supplementary figures and images for: Needle Tip Detection Using Ultrasound Probe for Vertical Punctures: A Simulation and Experimental Study
Source: Diagnostics (Basel). 2022 Feb 18;12(2):527. doi: 10.3390/diagnostics12020527 (PMC8871038; doi:10.3390/diagnostics12020527)

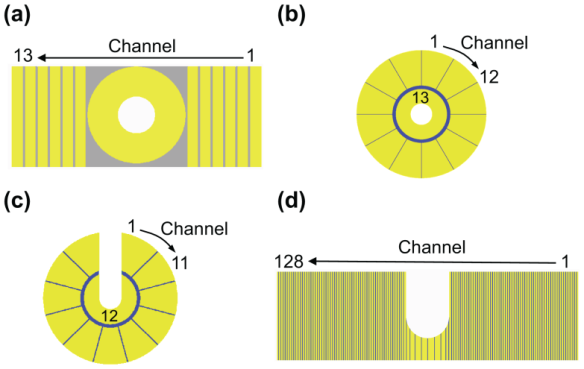

Supplement: Supplementary file 1 [file diagnostics-12-00527-s001.zip › diagnostics-1406553-supplementary/Manuscript-Supplementary/Supplement_Figure/Figure S1.tif]

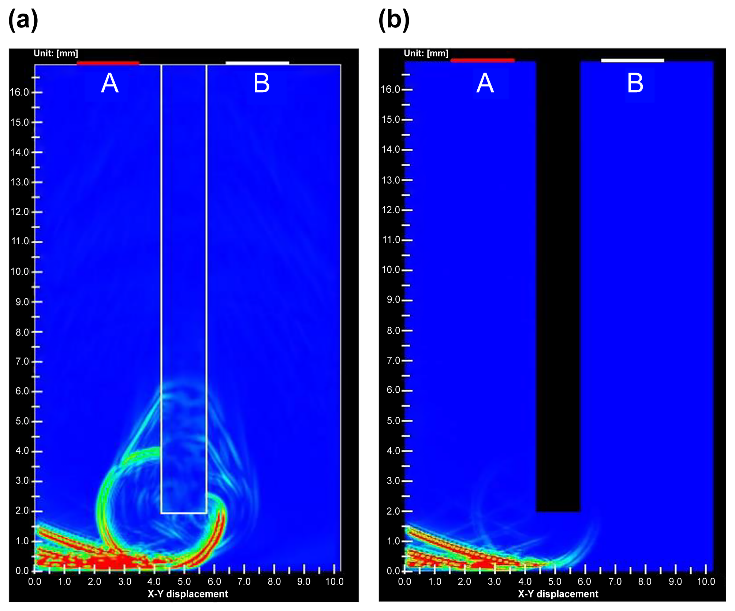

Supplement: Supplementary file 1 [file diagnostics-12-00527-s001.zip › diagnostics-1406553-supplementary/Manuscript-Supplementary/Supplement_Figure/Figure S10.tif]

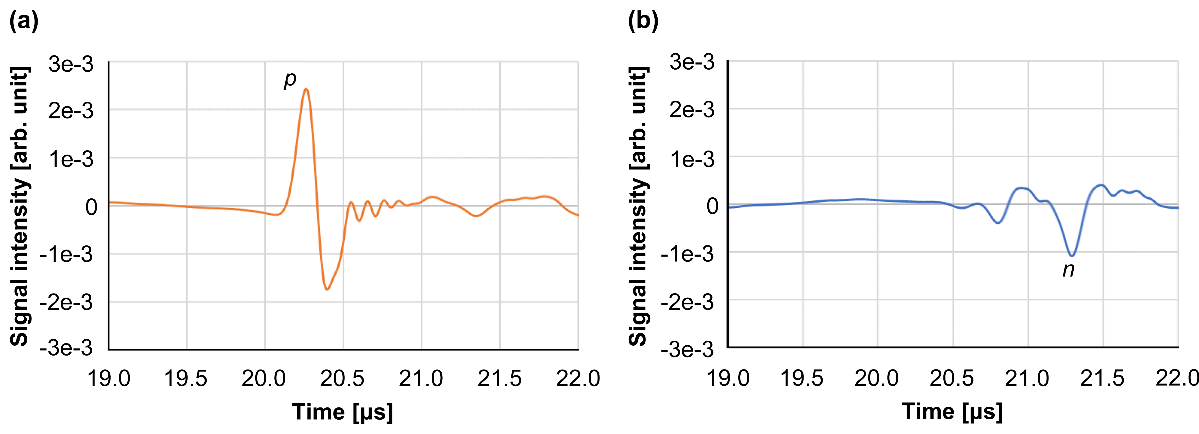

Supplement: Supplementary file 1 [file diagnostics-12-00527-s001.zip › diagnostics-1406553-supplementary/Manuscript-Supplementary/Supplement_Figure/Figure S11.tif]

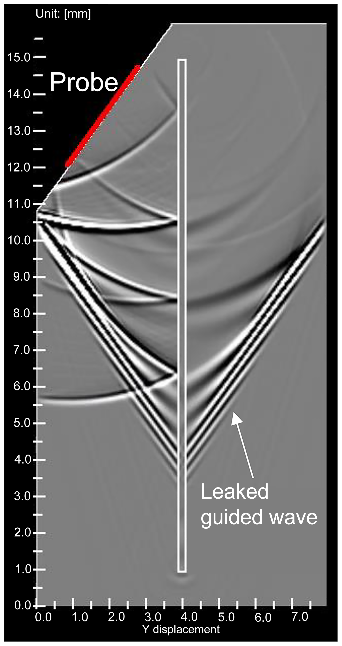

Supplement: Supplementary file 1 [file diagnostics-12-00527-s001.zip › diagnostics-1406553-supplementary/Manuscript-Supplementary/Supplement_Figure/Figure S12.tif]

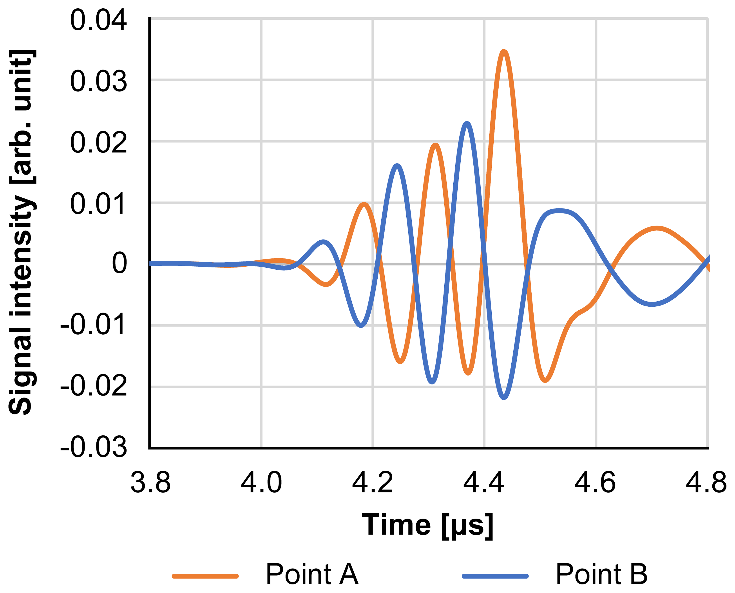

Supplement: Supplementary file 1 [file diagnostics-12-00527-s001.zip › diagnostics-1406553-supplementary/Manuscript-Supplementary/Supplement_Figure/Figure S13.tif]

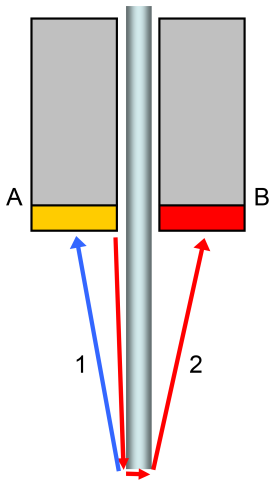

Supplement: Supplementary file 1 [file diagnostics-12-00527-s001.zip › diagnostics-1406553-supplementary/Manuscript-Supplementary/Supplement_Figure/Figure S14.tif]

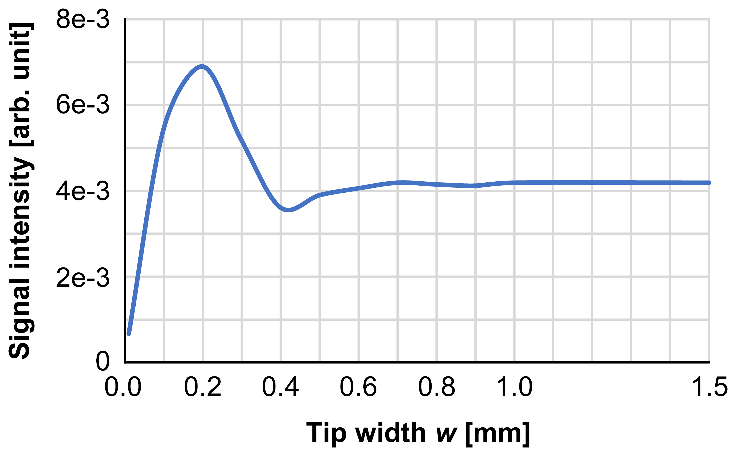

Supplement: Supplementary file 1 [file diagnostics-12-00527-s001.zip › diagnostics-1406553-supplementary/Manuscript-Supplementary/Supplement_Figure/Figure S15.tif]

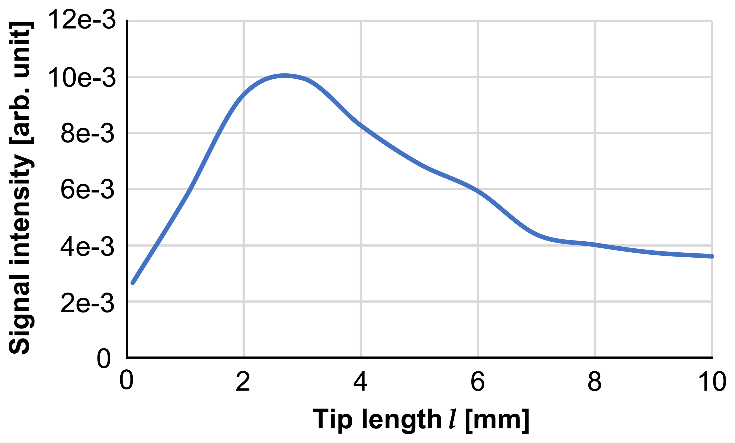

Supplement: Supplementary file 1 [file diagnostics-12-00527-s001.zip › diagnostics-1406553-supplementary/Manuscript-Supplementary/Supplement_Figure/Figure S16.tif]

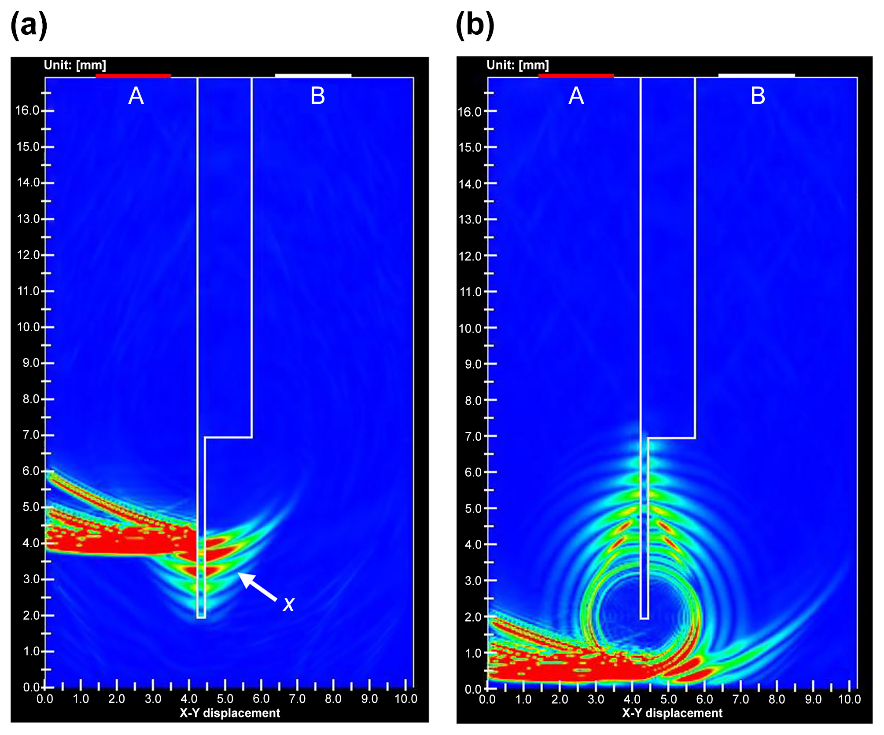

Supplement: Supplementary file 1 [file diagnostics-12-00527-s001.zip › diagnostics-1406553-supplementary/Manuscript-Supplementary/Supplement_Figure/Figure S17.tif]

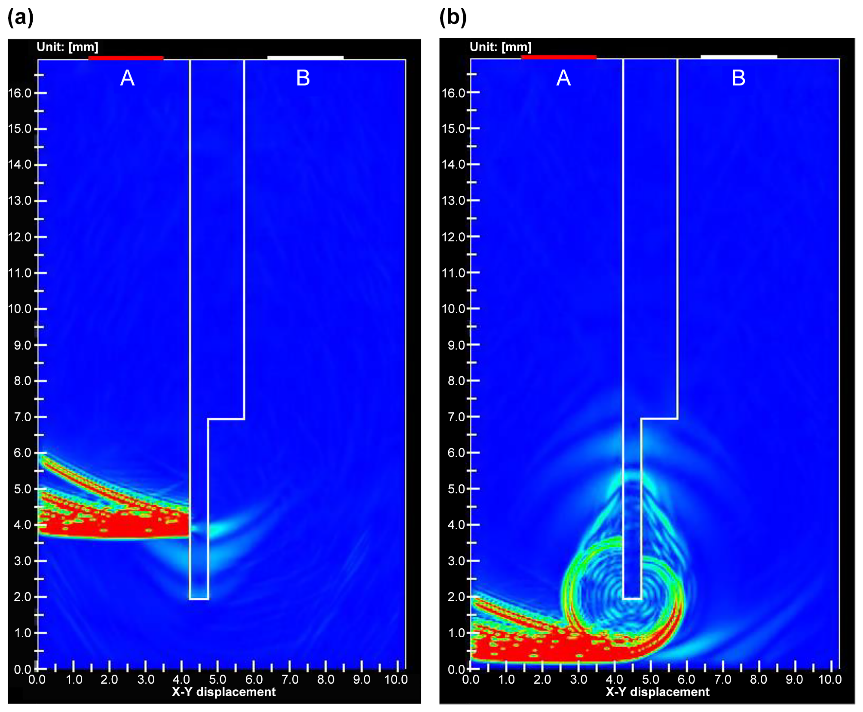

Supplement: Supplementary file 1 [file diagnostics-12-00527-s001.zip › diagnostics-1406553-supplementary/Manuscript-Supplementary/Supplement_Figure/Figure S18.tif]

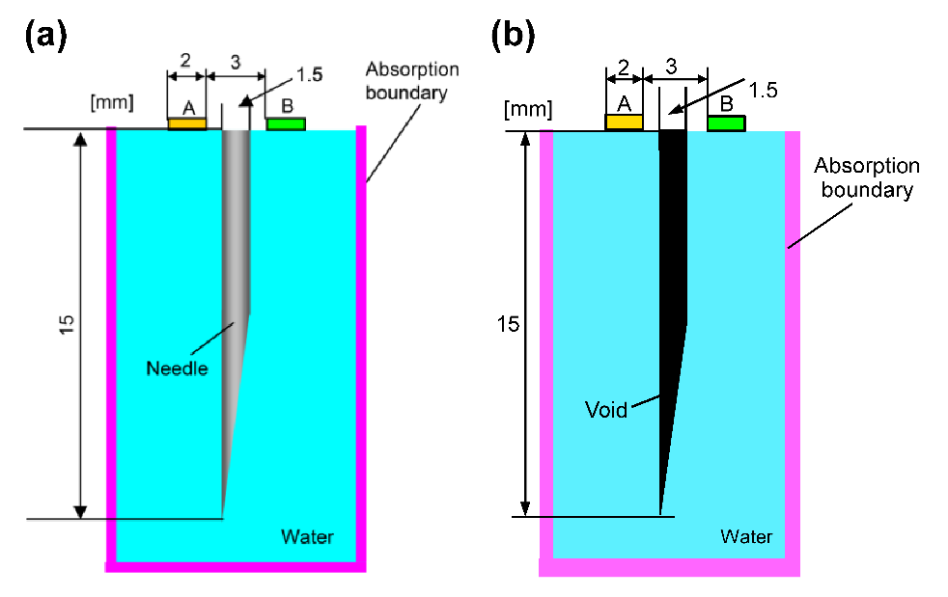

Supplement: Supplementary file 1 [file diagnostics-12-00527-s001.zip › diagnostics-1406553-supplementary/Manuscript-Supplementary/Supplement_Figure/Figure S2.tif]

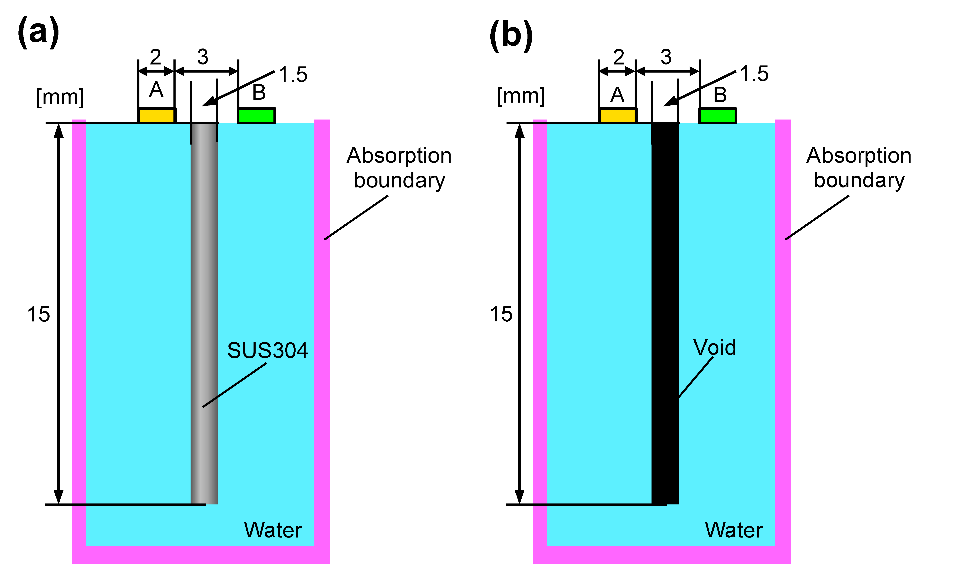

Supplement: Supplementary file 1 [file diagnostics-12-00527-s001.zip › diagnostics-1406553-supplementary/Manuscript-Supplementary/Supplement_Figure/Figure S3.tif]

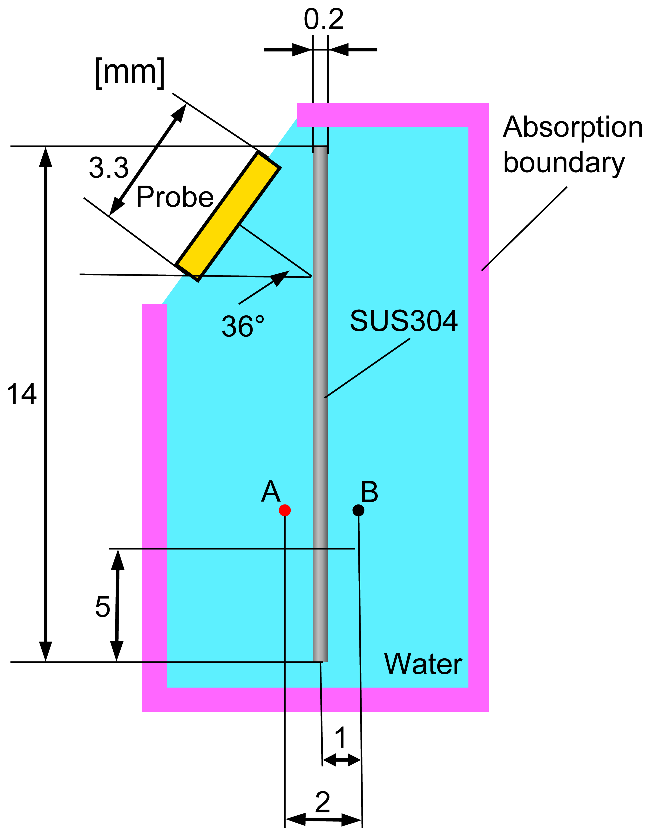

Supplement: Supplementary file 1 [file diagnostics-12-00527-s001.zip › diagnostics-1406553-supplementary/Manuscript-Supplementary/Supplement_Figure/Figure S4.tif]

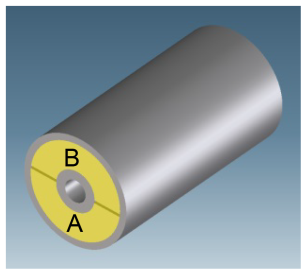

Supplement: Supplementary file 1 [file diagnostics-12-00527-s001.zip › diagnostics-1406553-supplementary/Manuscript-Supplementary/Supplement_Figure/Figure S5.tif]

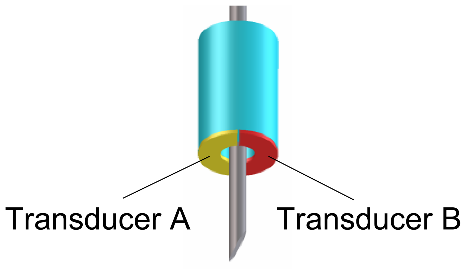

Supplement: Supplementary file 1 [file diagnostics-12-00527-s001.zip › diagnostics-1406553-supplementary/Manuscript-Supplementary/Supplement_Figure/Figure S6.tif]

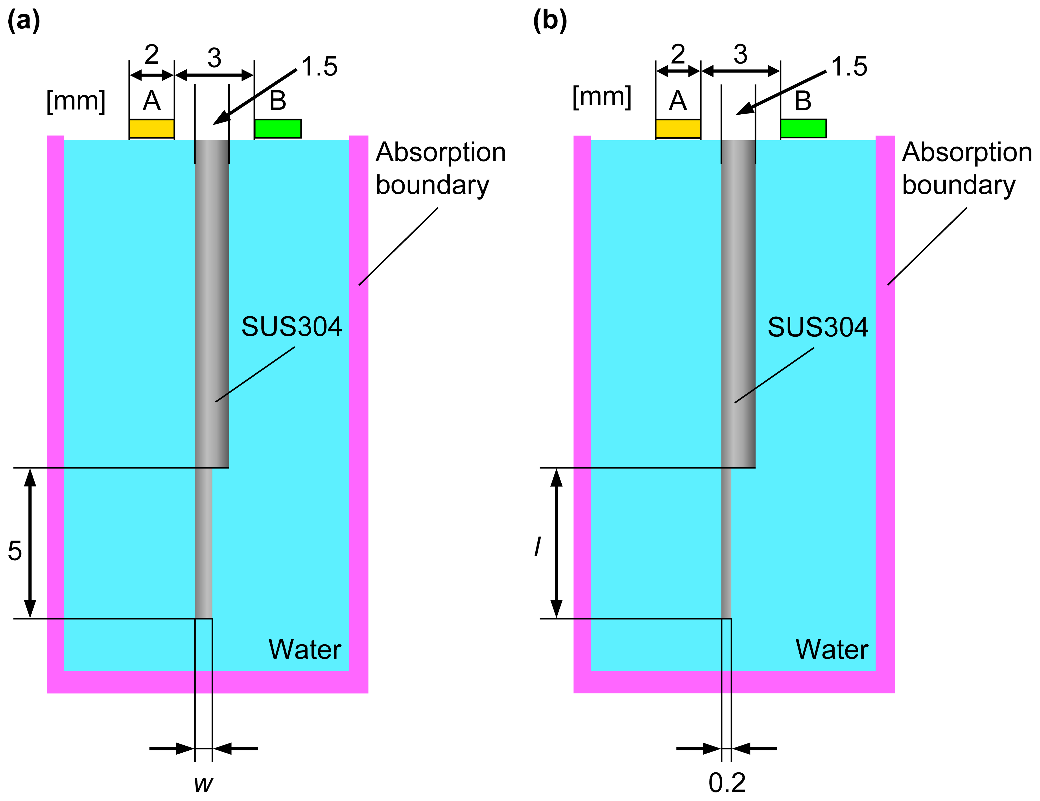

Supplement: Supplementary file 1 [file diagnostics-12-00527-s001.zip › diagnostics-1406553-supplementary/Manuscript-Supplementary/Supplement_Figure/Figure S7.tif]

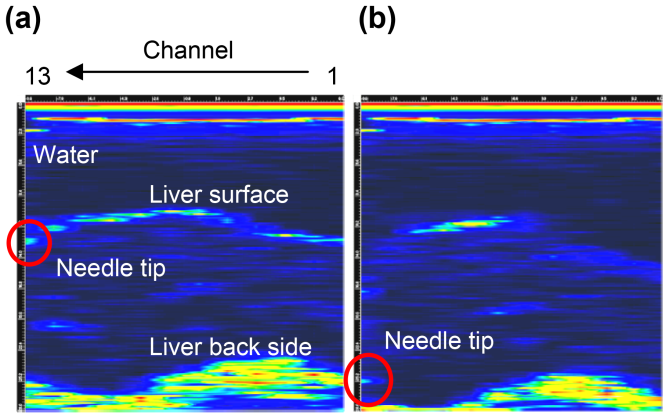

Supplement: Supplementary file 1 [file diagnostics-12-00527-s001.zip › diagnostics-1406553-supplementary/Manuscript-Supplementary/Supplement_Figure/Figure S8.tif]

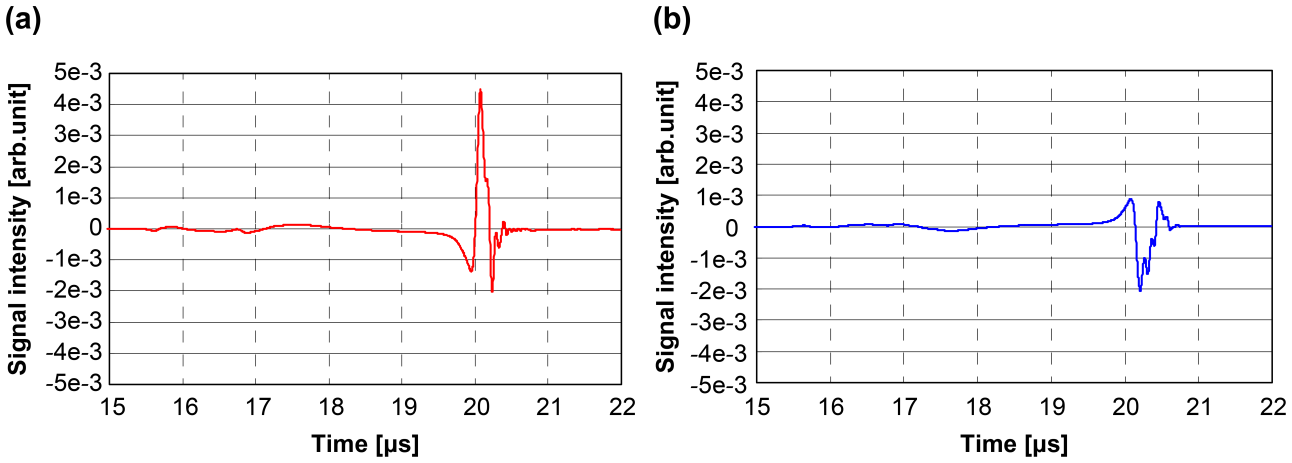

Supplement: Supplementary file 1 [file diagnostics-12-00527-s001.zip › diagnostics-1406553-supplementary/Manuscript-Supplementary/Supplement_Figure/Figure S9.tif]
